# Spatial distribution of HD-EMG improves identification of task and force in patients with incomplete spinal cord injury

**DOI:** 10.1186/s12984-016-0151-8

**Published:** 2016-04-29

**Authors:** Mislav Jordanic, Mónica Rojas-Martínez, Miguel Angel Mañanas, Joan Francesc Alonso

**Affiliations:** Department of Automatic Control (ESAII), Biomedical Engineering Research Centre (CREB), Technical University of Catalonia UPC, Barcelona, Spain; Biomedical Research Networking Center in Bioengineering, Biomaterials and Nanomedicine (CIBER-BBN), Barcelona, Spain

**Keywords:** Myoelectric control, Pattern recognition, High-density electromyography, Incomplete spinal cord injury

## Abstract

**Background:**

Recent studies show that spatial distribution of High Density surface EMG maps (HD-EMG) improves the identification of tasks and their corresponding contraction levels. However, in patients with incomplete spinal cord injury (iSCI), some nerves that control muscles are damaged, leaving some muscle parts without an innervation. Therefore, HD-EMG maps in patients with iSCI are affected by the injury and they can be different for every patient. The objective of this study is to investigate the spatial distribution of intensity in HD-EMG recordings to distinguish co-activation patterns for different tasks and effort levels in patients with iSCI. These patterns are evaluated to be used for extraction of motion intention.

**Method:**

HD-EMG was recorded in patients during four isometric tasks of the forearm at three different effort levels. A linear discriminant classifier based on intensity and spatial features of HD-EMG maps of five upper-limb muscles was used to identify the attempted tasks. Task and force identification were evaluated for each patient individually, and the reliability of the identification was tested with respect to muscle fatigue and time interval between training and identification.

**Results:**

Three feature sets were analyzed in the identification: 1) intensity of the HD-EMG map, 2) intensity and center of gravity of HD-EMG maps and 3) intensity of a single differential EMG channel (gold standard). Results show that the combination of intensity and spatial features in classification identifies tasks and effort levels properly (Acc = 98.8 %; S = 92.5 %; P = 93.2 %; SP = 99.4 %) and outperforms significantly the other two feature sets (*p* < 0.05).

**Conclusion:**

In spite of the limited motor functionality, a specific co-activation pattern for each patient exists for both intensity, and spatial distribution of myoelectric activity. The spatial distribution is less sensitive than intensity to myoelectric changes that occur due to fatigue, and other time-dependent influences.

## Background

Surface electromyography (sEMG) is commonly used in noninvasive extraction of motor control information and identification of motion intention. Therefore, it has a wide practical application in rehabilitation engineering, e.g., prosthetics [1–3], exoskeletons [4] and rehabilitation robots [5, 6].

Conventional myocontrol is based on non-pattern recognition strategies. In a classical example of a single joint prosthesis (one degree of freedom), sEMG signals are recorded on two independent muscles. EMG of one muscle controls the intensity in one movement direction, and the EMG of another muscle in the opposite direction. The output force is proportional to EMG power of the controlling muscle. This strategy is simple, computationally efficient, robust, and does not need training, which makes it suitable for unsupervised, everyday use. However, it allows control only in one degree of freedom (DoF) at a time. Although this approach can provide intuitive interface with fewer commands [7], in case of a prosthetic device with multiple degrees of freedom (e.g. hand prostheses), switching between DoFs is impractical and requires a long time to complete a complex task [8].

On the other hand, pattern recognition-based control strategy enables usage of multiple DoFs without switching, which significantly improves task completion time [7]. Although a variety of classifiers (e.g. hidden Markov model, support vector machine, artificial neural network, fuzzy logic) have been evaluated for task identification [9], multiple authors agree that the identification does not significantly depend on the classifier type [7, 10, 11]. Therefore, simple and easy to train classifiers, e.g. linear discriminant analysis (LDA), are preferred [12–15]. Conversely, finding an appropriate set of features is challenging [16–19]. Time-domain features are commonly used because they can achieve high identification results and are computationally efficient [7].

The technological advancement of EMG acquisition systems [20, 21] enables the use of high-density electromyography (HD-EMG). By using an array of closely spaced electrodes organized in a quadrature grid, a wide muscle area is recorded. This technology allows insights into the spatial distribution of the myoelectric intensity of a muscle. The spatial distribution allows monitoring the activation of different muscle regions, which depends on joint position [22], contraction level [23], and duration of movement [24]. In addition, it has already been reported that spatial features can be used in task identification in normal subjects [3, 25].

In patients with neurological disorders (e.g., stroke, spinal cord injury) motor control is impaired and some muscle parts can be left without innervation. As a result, patients often have problems with uncoordinated movements, lack of force, and spasticity. Rehabilitation and therapy can partially regenerate motor control, and either the affected muscles can recover partial functionality or other muscle groups can replace the functionality of a dysfunctional part. Therefore, the spatial distribution of motor unit action potentials is different from subject to subject and depends on the injury. But is it task-specific? And a more interesting question: is it force-specific? Liu & Zhou [17] already proved that an intensity-related muscle co-activation pattern exists and that different hand tasks can be successfully identified in patients with incomplete spinal cord injury (iSCI). But can spatial distribution of myoelectric intensity help in identification of task and level of effort in patients with iSCI?

In this work, a method for the identification of different tasks and effort levels in patients with iSCI is proposed. High density EMG was measured on muscles participating in the analyzed contractions. By using different feature sets and an LDA classifier, we demonstrate that a specific co-activation pattern exists in patients with iSCI not only for a certain task, but also for a contraction intensity. Furthermore, the influence of time-dependent changes in EMG signal (due to muscle fatigue and drying of conductive gel) on the reliability of identification was evaluated. It was demonstrated that features related to spatial distribution not only improve the identification, but they are also more robust to time changes. What is more, they are helpful when identifying both the task and the desired force, indicating that spatial activation of motor units depends on type of exercise and contraction level in patients with iSCI.

## Method

### Measurements

#### Instrumentation

**Fig. 1:**
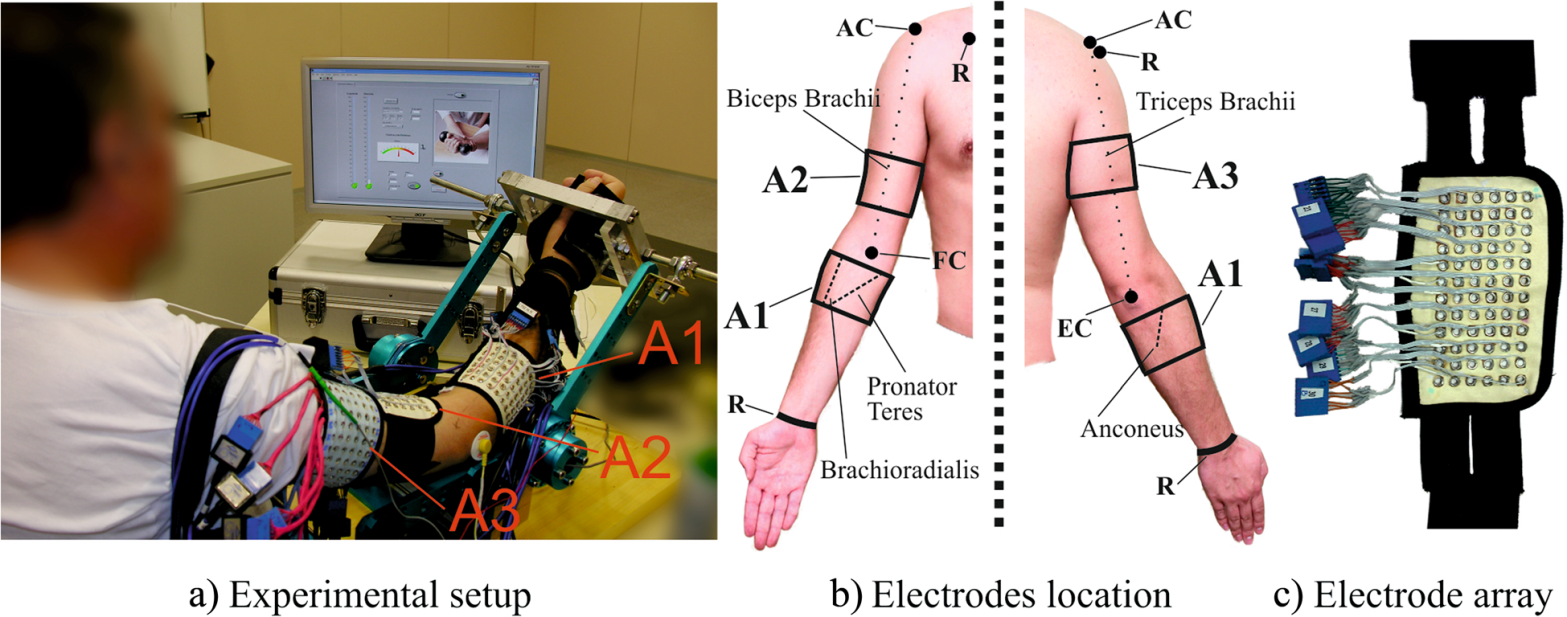
Experimental setup. **a** Positioning of the electrode arrays A1-A3 during the recording. **b** Anatomical landmarks and paths used for the positioning of the arrays: A1 (6 rows, 16 columns) was placed over the forearm covering Anconeus, Brachioradialis and Pronator Teres muscles, where the most proximal row of electrodes was placed ~2 cm bellow the elbow crest (EC) covering all three muscles, according to [46]; A2 (6 rows, 12 columns) was placed in the distal part of the upper arm with respect to the center of the line connecting fossa cubit (FC) and acromion (AC), and covering Biceps Brachii muscle; A3 (6 rows, 12 columns) was placed in the proximal part with respect to the center of the line connecting EC and AC, over Triceps Brachii. Both A2 and A3 arrays were located in accordance with SENIAM recommendations [33]. Reference electrodes (R) were placed on the clavicle, wrist and shoulder of the active arm. **c** Detail of the electrode arrays used in the experiment

For the recording of HD-EMG signals, 2-D electrode arrays were fabricated in our laboratory (see Fig. 1c). They were designed as silver-plated eyelets (5 mm external diameter), embedded in a hydrophobic fabric in a quadrature grid with 10 mm inter-electrode distance. When positioned and fixed with elastic straps, fabric follows the contour of the muscle enabling a constant electrical contact between subject’s skin and eyelets.

In total, 240 monopolar EMG channels were recorded for each patient using three electrode arrays. A “driven right leg” circuit [26] was used to reduce the common mode interference by feeding the common mode voltage with opposite phase to the patient.

Monopolar EMG signals were digitized using two amplifiers with synchronized sampling (EMG-USB- 128 channels, sampling frequency 2048 Hz, 3 dB bandwidth 10–750 Hz, programmable gains of 100, 200, 500, 1000, 2000, 5000, 10000, manufactured by LISiN-OT Bioelettronica).

In order to perform isometric contractions at the desired force, a mechanical brace was used and torque transducers (OT Bioelettronica, range 150 Nm, resolution 2.5 mV/V) were placed on each joint to record the exerted torque (Fig. 1). During the measurements, patients were sitting upright in front of the brace with their dominant arm immobilized at the wrist to avoid hand grip. The forearm was in the sagittal plane, halfway between pronation and supination. The elbow was flexed at 45° and the shoulder was adducted at 90° in the horizontal plane and flexed at 45° in the sagittal plane. The exerted force level was displayed online to patients during the exercise for visual feedback.

#### Experimental setup

Nine patients (four male, five female; age: 47 ± 18 years; body mass index: 28.2 ± 4.2) diagnosed with iSCI at C4-C6 levels participated in the study. Patients were rated C or D according to the ASIA scale and were injured at least 1 month before the experimental session. The study was conducted in accordance with the Declaration of Helsinki and subsequent amendments concerning research in humans and was approved by the Hospital Ethics Committee and the Local Government. All volunteers gave their written informed consent to participate.

HD-EMG was recorded during four isometric upper-limb tasks, i.e. flexion/extension of the elbow and supination/pronation of the forearm, on five superficial muscles involved by these tasks: Biceps Brachii, Triceps Brachii, Anconeus, Brachioradialis, and Pronator Teres. Prior to positioning of the electrode arrays, skin was cleaned, shaved, and treated with abrasive gel.

Three electrode arrays were used during the experiment: array **A1** was placed over the forearm covering Anconeus, Brachioradialis and Pronator Teres muscles, and arrays **A2** and **A3** were placed over the upper arm covering Biceps Brachii and Triceps Brachii muscles. Reference electrodes were placed on the clavicle, wrist and shoulder of the active arm. After placing the arrays, each eyelet was filled with 20 μl of conductive gel using a gel dispenser (Multipette Plus, Eppendorf, Germany). The experimental setup can be seen in Fig. 1.

#### HD-EMG recordings

Before signal recording, the maximal voluntary contraction (MVC) was measured for each task as a maximum of three consecutive trials. To prevent fatigue, each trial was followed by a three minute rest [27, 28]. Patients were trained to keep their fingers and wrist relaxed in order to minimize the activity of forearm muscles that do not participate in the intended tasks.

The measurement protocol was composed of two parts. In the first part, contractions at three levels of effort (10 %, 30 % and 50 % MVC) were measured for each task in randomized order. Visual feedback of the level of effort was provided in real time and subjects were asked to maintain the target level as precise as possible. Patients were instructed to remain at rest for three seconds followed by a contraction at a predefined force level for 10 s. There were three-minute breaks between consecutive recordings to prevent cumulative fatigue.

The second part of the measurement protocol began approximately half an hour (27.0 ± 9.8 min) after the end of the first part of the protocol. Each measurement started with a three-second rest period after which patients performed contraction at 50 % MVC until failure. The procedure was repeated for each task and between recordings there were three-minute breaks.

**Fig. 2:**
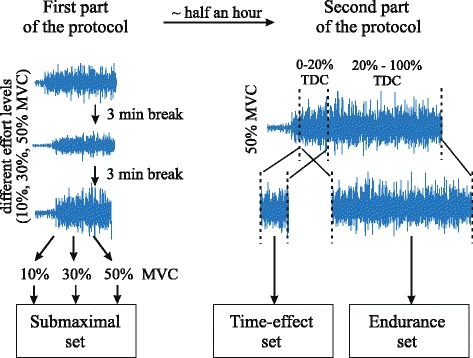
HD-EMG recording flow chart: Flow chart describes recording protocol of each task. Note that the recordings order was randomly selected in each part of the protocol

The recorded signals were divided into three sets for the subsequent analysis: the first set (submaximal set) was composed of the signals recorded in the first part of the protocol. The second set (time-effect set), used to test the time effect on the identification, was extracted from the beginning (up to 20 % of the total duration of the contraction, TDC) of the signals recorded in the second part of the protocol. Finally, the third set (endurance set) was used to test the effect of myoelectric fatigue on the identification, and was composed of the totality of the signals recorded in the second part of the protocol. The flow chart of the recording protocol can be seen in Fig. 2.

### HD-EMG maps and feature extraction

#### HD-EMG maps calculation

Low quality channels, a common issue in HD-EMG measurements, were identified by an expert system proposed by Rojas-Martínez et al. [29]. The system is based on thresholds associated with the following three features: 1) relative power of low frequency components (from 0 to 12 Hz); 2) relative power of power-line components (50 Hz and first four harmonics); and 3) power calculated from RMS value of the signal. EMG channels without measurement artifacts were zero-phase filtered between 15 Hz and 350 Hz (Butterworth bandpass filter, 4th order), and the first 6 harmonics of power line coupling were suppressed by using the adaptive transversal filter described in [30], whose weights were estimated by a least mean squares algorithm.

HD-EMG maps represent the spatial distribution of intensities of active motor units over the surface of the muscle:

1 $$ H{M}_{i,j}=RMS\left(sEM{G}_{i,j}\right) $$

where *HM* is an activation map and each pixel in a map (*HM*_*i,j*_) corresponds to an RMS value of a channel in an electrode array (position *i,j*). Maps were calculated on non-overlapping time windows of 250 ms to ensure an acceptable response time in applications directed to myoelectric control [9], and channels previously identified as artifacts were replaced by triangle-based cubic interpolation [29].

#### Feature extraction

Two types of features related to HD-EMG maps were extracted: intensity and center of gravity. They were used in classification individually or combined in order to compare their performance. Additionally, the intensity of a single differential channel, i.e. traditional bipolar recordings usually employed in pattern recognition as a “gold standard”, was compared to other features. In any case, the feature set was composed of features extracted from all 5 monitored muscles.

Multiple studies suggest that the relationship between EMG amplitude and generated force is not linear [31, 32]. Accordingly, the intensity features were calculated as a common logarithm of the mean intensity of the HD-EMG maps, which proved to achieve higher classification results than a linear measure [25]:

2 $$ I={ \log}_{10}\frac{1}{N}{\displaystyle \sum_{i,j}}H{M}_{i,j} $$

where *I* is an intensity feature calculated from the HD-EMG intensity map *HM* with a total number of *N* channels, and *HM*_*ij*_ is the intensity of a channel located at position *i,j*.

The center of gravity of an HD-EMG (*CG*) map was calculated as:

3 $$ CG=\frac{1}{{\displaystyle {\sum}_{i,j}}H{M}_{i,j}}{\displaystyle \sum_{i,j}}H{M}_{i,j}\ \left[\begin{array}{c}\hfill i\hfill \\ {}\hfill j\hfill \end{array}\right] $$

where (*i,j*) represents a channel position in the HD-EMG map *HM*.

The intensity of a single differential channel (*Diff*) was calculated as a common logarithm of an RMS value of difference of two consecutive channels in the direction of muscle fibers:

4 $$ Diff=lo{g}_{10}\left(RMS\left(sEM{G}_{i,j}-sEM{G}_{i+1,j}\right)\right) $$

where the locations of channels (*i,j*) and (*i + 1,j*) are selected following SENIAM recommendations [33]. *Diff* was calculated on the same 250 ms time epoch as the HD-EMG map.

### Identification of motion intention

#### Classification

Three LDA classifiers based on different feature sets extracted from all five monitored muscles were evaluated in the study:

1. Classifier based on the intensity of the HD-EMG map (I)
2. Classifier based on the intensity and center of gravity of the HD-EMG map (I + CG)
3. Classifier based on the intensity of a single differential channel (gold standard) (Diff)

These classifiers were evaluated in the identification of task and level of contraction in patients with iSCI. Furthermore, the reliability of the classifiers was tested with respect to the slow time-dependent changes occurring in myoelectric signals, like those associated with gel drying or those related to changes at the physiological level (myoelectric fatigue).

Available observations were divided into a training group, which was used to train the classifier, and a validation group, which was used to evaluate classifier’s performance. Both groups were balanced, i.e. there was an equal number of observations of each class in the training group, as well as in the validation group, and data were split into training and validation sets using a 50 % / 50 % ratio [34]. To confirm the model was not overfitted, the results of classification of both sets were compared and were found similar. To achieve the statistical stability of results, each classifier was trained and evaluated in one thousand iterations, which are enough to avoid the potential error due to bad data partitioning [35], and then classification results were averaged. In every iteration, observations in the training and validation groups were assigned randomly.

The performances of the classifiers were expressed in terms of accuracy (Acc), sensitivity (S), precision (P) and specificity (SP) [36], as described in the following equations:

5 $$ Acc=\frac{TP+TN}{TP+FP+TN+FN} $$

6 $$ S=\frac{TP}{TP+FN} $$

7 $$ P=\frac{TP}{TP+FP} $$

8 $$ SP=\frac{TN}{TN+FP} $$

where true positives (TP) is the number of samples correctly appended to a certain class; true negatives (TN) is the number of samples that do not belong to a certain class and were not classified to that class; false positives (FP) is the number of samples not belonging to a certain class, but wrongly classified into that class; and false negatives (FN) is the number of samples belonging to a certain class, but wrongly classified into another class.

#### Short-term identification

Classifiers with different sets of features (I, I + CG, and Diff) were tested on the submaximal set. Signals belonging to this set were recorded in a short time interval and, consequently, in the same conditions.

Two types of identification were considered: 1) Identification of tasks and 2) Identification of tasks and effort levels. **Identification of tasks** had 4 classes corresponding to the type of the task (flexion, extension, supination, and pronation) and an additional fifth class that corresponds to the rest period – no activity class (NoAct). Observations of no activity were extracted from the first three seconds of each recording, where subjects were asked to maintain at rest. Activity classes consisted of a mixture of all effort levels. On the other hand, **identification of tasks and effort levels** had 13 classes: 4 tasks with 3 levels of effort for each task (10 % MVC, 30 % MVC and 50 % MVC) and NoAct class.

Considering that patients were not always able to maintain the target level of contraction given their condition, the torque signal was used to select only time segments where the measured force remained within a threshold of ±5 %, ±10 % and ±10 % MVC for target contractions at 10 %, 30 % and 50 % MVC. From every submaximal contraction 20 non-overlapping, 250 ms time epochs, closest to the target force were selected. This procedure ensured 20 observations for each task with differentiation on the level of effort, or 60 samples for each task, without differentiation on the effort level. Consequently, 60 observations without muscle activity were selected for NoAct class from the beginnings of exercises (rest period).

#### Influence of time- progress on identification

Wet electrodes with conductive electrolytic gel are commonly used for sEMG recording. However, these electrodes are not good for long-term monitoring [37]. Gel drying increases skin-electrode impedance, affecting amplitude and spectral content of the recorded signal. Moreover, skin perspiration is enhanced under the electrode array, which also affects the skin-electrode impedance and, consequently, the characteristics of the recorded signal. To compare the performances of the different features, task identification was tested in these conditions.

Classifiers were trained on the submaximal set and validated on the time-effect contractions recorded in the second part of the protocol. As in the previous section, 20 time epochs for each task and level of effort were identified from the submaximal set based on the torque signal. Half the extracted observations of all levels of effort were used for training, following the recommendations of Scheme and Englehart [12], where it was noticed that a mixture of effort levels in the training group yields a more robust classifier. NoAct observations for the training group were extracted from recordings in the first part of the measurement protocol, whereas observations for the validation group were extracted from recordings in the second part of the protocol.

For comparison, the same classifier was used to validate contractions recorded at the first part of the protocol, i.e. using samples of the submaximal set. Since the classifier was trained on just half of the available observations from the submaximal set, the remaining observations were used for validation. But considering that time-effect set was composed of contractions recorded at 50 % MVC effort level, the validation group was also composed only of 50 % MVC contractions from the submaximal set.

The classifier was trained and evaluated over 1000 iterations with observations selected randomly both in the training and validation sets to avoid bias in the performance.

#### Influence of muscle fatigue on identification

Muscle fatigue is a slow change that occurs in contracting muscles. It alters the characteristics of recorded sEMG signal (i.e. amplitude and frequency content) [38] and, inherently, alters the extracted classification features [39]. To test the effect of fatigue on identification, each recording in the endurance set was divided into five equal time segments, i.e. 0–20 % TDC, 20–40 % TDC, 40–60 % TDC, 60–80 % TDC, and 80–100 % TDC. The first segments (0–20 % TDC) were used as a training group and the identification was carried out on all segments. The classification indices (accuracy, sensitivity, precision and specificity) were calculated for each segment in order to monitor performance during fatigue. The number of observations of each class was the same in the training group, as well as in the validation group.

#### Statistical methods

A repeated measures analysis of variance (ANOVA) was applied to the different performance indices using each type of task and effort level as measures and features used in the classification as factors. Both, within-subject and between-subject effects were considered in the analysis. In the case of endurance analysis, the repeated measures test was applied to account for differences attributed to the factor time, that is, duration of the contraction. In addition, differences between means were assessed through Student’s *t*-test for paired samples. Effects and differences were considered significant at *p* = 0.05.

## Results

### Short-term identification

The different combinations of feature sets extracted from the five recorded muscles (l, I + CG, Diff) were evaluated in non-changing conditions, i.e. training and validation groups were extracted from the same contractions (submaximal set). Features were evaluated in 2 types of identification: 1) identification of tasks and 2) identification of tasks and effort levels.

**Fig. 3:**
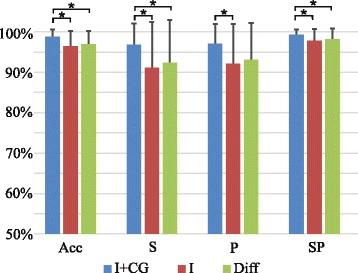
Identification of tasks: Average classification indices (Acc, S, P, SP) are shown for classifiers based on different sets of features (I + CG, I, and Diff). Symbol ”*” indicates statistical significance (*p* < 0.05)

The results of task identification are shown in Fig. 3. Adding spatial features to the classification improves the results and decreases the standard deviation. This is especially pronounced in sensitivity of flexion (88,8 % ± 12,6 % and 96,7 % ± 5,5 % in mean and standard deviation for I and I + CG features, respectively) and extension (89,6 % ± 12,1 % and 98,7 % ± 2,0 % for I and I + CG features, respectively) as well as in precision of pronation (89,9 % ± 12,5 % and 96,6 % ± 6,3 % for I and I + CG features, respectively), and NoAct (85,6 % ± 15,3 % and 94,8 % ± 6,5 % for I and I + CG features, respectively). When evaluating differences in the performance of features through the repeated measures ANOVA, the within-subject effect was not significant when comparing indices obtained with the feature I or with the combination of features I + CG (either for accuracy, sensitivity, precision or specificity). However, the between subject effect was significant (*p* < 0.05 in all cases), showing that performance obtained for the combination of features I + CG was higher than that obtained when using the features I in the classification, independently of the evaluated task. Similar results were obtained when comparing performance of features Diff and I + CG: the within-subject effect showed no significant differences, that is, similar indices were obtained for all tasks (flexion, extension, supination, pronation and no activity), while the between-subjects effect was significant for all indices (*p* < 0.05) except for precision (*p* = 0.07), showing a higher performance for the features I + CG. No significant effects were observed when comparing the performance indices obtained with the features I with those obtained with the features Diff (p.n.s.).

**Fig. 4:**
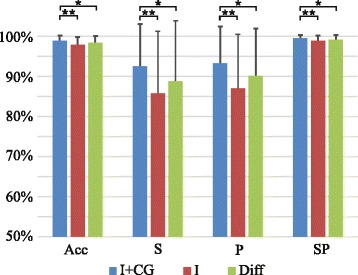
Joint identification of tasks and effort levels: Average classification indices (Acc, S, P, SP) are shown for classifiers based on different sets of features (I + CG, I, and Diff). Symbols ”*” and “**” indicate statistical significance *p* < 0.05 and *p* < 0.01, respectively

Figure 4 shows the results of identification of tasks and effort levels. It can be noticed from the results that the identification based on intensity and spatial features displayed, in average, higher performance and lower standard deviation than the other two classifiers. Like in the previous case, the within-subject effect when comparing either between performance indices of I and I + CG or between performances of Diff and I + CG was not significant, showing similar results for all 13 classes (tasks and effort levels and no activity). However, the between-subjects effect was significant in both analyses (*p* < 0.001 when comparing I and I + CG; *p* < 0.02 when comparing Diff and I + CG), showing a higher performance for the case of the combination I + CG. Finally, when comparing performances between features I and Diff, no significant effects were observed (p.n.s.).

**Fig. 5:**
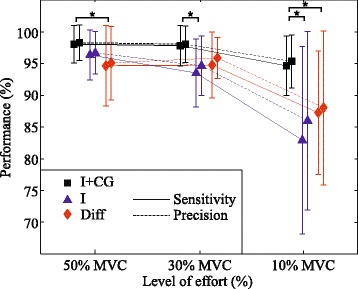
Identification of tasks at specific levels of effort: Sensitivity and precision are shown for classifiers based on different sets of features (I + CG, I, Diff). Each classifier was trained using contractions of all effort levels, and evaluated on contractions of specific effort levels. Symbol ”*” indicates statistical significance (*p* < 0.05.)

Figure 5 shows the performance of identification of tasks performed at a specific effort level. In this case, the classifier was trained using a mixture of all effort levels. The training group and the validation group were both extracted from the submaximal set. It can be noticed that all feature sets performed well when identifying tasks corresponding to high levels of contraction, but only the identification with spatial distribution maintained high performance and low standard deviation even at low contraction levels, i.e. 10 % MVC, where paired t-tests showed that the identification based on intensity and spatial features significantly outperforms the other two types of features (*p* < 0.04).

### Influence of time on identification

**Fig. 6:**
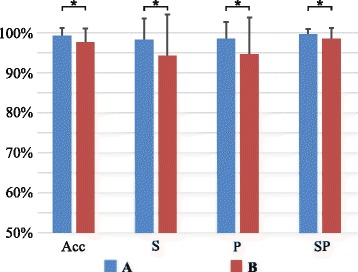
Time influence on the identification of tasks: Average classification indices (Acc, S, P, SP) are shown for the classifier based on the I + CG features. In blue bars ”A”, training and validation sets were recorded during the first part of the protocol, whereas in red bars ”B” training and validation sets were recorded during first and second part of the protocol, respectively. Symbol ”*” indicates statistical significance (*p* < 0.05)

For the purpose of evaluation of the effect of time on identification, a classifier based on I + CG was trained using the submaximal set, and the identification was tested both on the submaximal set, and the time-effect set. Results are shown in Fig. 6, where it is possible to observe that the average performance significantly decreased with time (paired samples *t*-test showed *p* < 0.05) whereas the standard deviation increased.

**Fig. 7:**
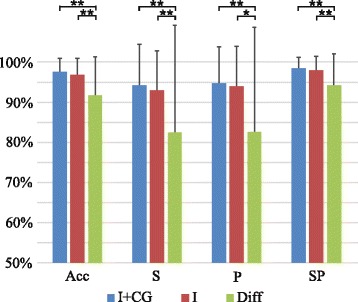
Influence of time effect on the identification: Figure shows average classification indices (Acc, S, P, SP) for classifiers based on different feature sets (I + CG, I, Diff). Training set was recorded during the first part of the protocol, and the validation set was recorded during the second part of the protocol. Symbols ”*” and “**” indicate statistical significance *p* < 0.05 and *p* < 0.01, respectively

Figure 7 shows performances of the different feature sets when the validation group was recorded after the training group, i.e. the classifier was trained on the submaximal set and recorded on the time-effect set. It can be noticed that the identification based on Diff features exhibited a significantly lower performance than the identifications based on I or I + CG features (paired samples *t*-test showed *p* < 0.05), while the identification based on I features performed similarly to the identification based on I + CG features (p.n.s.). This last can be understood in light of the results presented in the previous section, where the identification performances using these feature sets were similar at high-middle levels of effort, but I + CG outperformed I features at low effort levels (see Fig. 5).

### Influence of muscle fatigue on identification

**Fig. 8:**
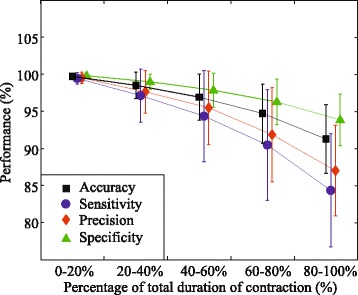
Fatigue influence on identification based on I + CG feature set: Average classification indices (Acc, S, P, SP) are shown along the endurance contraction for the classifier based on the I + CG feature set

Figure 8 shows the influence of muscle fatigue on the identification based on intensity and center of gravity of the HD-EMG maps. It can be observed that average classification indices gradually decrease with fatigue. When evaluating differences in the performance of these indices, the within-subject effect given by the repeated measures analysis was significant (*p* < 0,001 in all indices). This result relies on the assumption of sphericity, that is, variances of the differences between all pairs of the repeated measurements should be equal; otherwise, result is positively biased. The conservative Greenhouse-Geisser correction method for the lack of sphericity [40] was applied to adjust the degrees of freedom [41, 42] when the assumption of sphericity was violated. As suggested by Landa and Everitt [41], Mauchly’s test was used to test the sphericity.

**Fig. 9:**
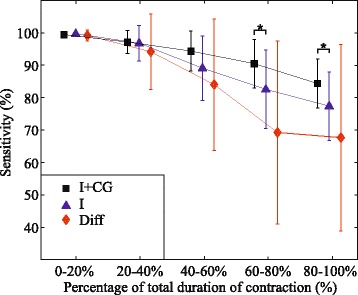
Fatigue influence on sensitivity using different sets of features: Average sensitivity along the endurance contraction is shown for classifiers based on different sets of features (I + CG, I, Diff). Symbol ”*” indicates statistical significance *p* < 0.05

**Fig. 10:**
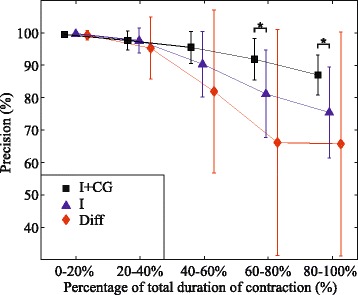
Fatigue influence on precision using different sets of features: Average precision along the endurance contraction is shown for classifiers based on different sets of features (I + CG, I, Diff). Symbol ”*” indicates statistical significance *p* < 0.05.”

Figures 9 and 10 display the influence of muscle fatigue on sensitivity and precision of the identification based on different feature sets. It can be noticed that all classifiers achieved high sensitivity and precision at the beginning of the endurance contractions, however, as the manifestations of myoelectric fatigue became more evident, the classifier based on intensity and spatial features outperformed the other two, both in average performance and variability.

## Discussion

Nine subjects with iSCI performed four isometric forearm tasks (flexion, extension, supination, and pronation) at three levels of effort (10 % MVC, 30 % MVC, and 50 % MVC). High density EMG was measured on five muscles of forearm and upper arm in monopolar configuration. Intensity maps were calculated for each muscle and three different feature sets were extracted: the average intensity of an HD-EMG map (I), the intensity and center of gravity of an HD-EMG maps (I + CG), and the intensity of a single differential channel (Diff) (gold standard). Using the extracted feature sets and LDA-based classification, both task and effort level were identified, and the influence of fatigue and other time-dependent changes (e.g. drying of conductive gel) on identification was evaluated. Since the goal of this study was to analyze different feature sets rather than classification methods, LDA was utilized given that this method is the most commonly used, and is generally recommended for myoelectric interfaces [7]. Although it assumes normal distribution of patterns in each class, it has proven to have good performance even when the normality assumption does not hold [43].

When identification using the different features was tested on signals recorded in short time intervals, the combination of I + CG outperformed the other feature sets. The results show that a muscular co-activation pattern exists not only for the task intention (Acc = 98.7 %; S = 96.8 %; P = 97.0 %; SP = 99.2 %), but also for the force intention (Acc = 98.8 %; S = 92.5 %; P = 93.2 %; SP = 99.4 %).

Although the identification based on the features Diff has slightly better performance in average than the identification based on the features I, a repeated measures ANOVA showed that there is no significant difference in their distributions. Moreover, a small displacement in the position of bipolar electrodes can have a great effect on signal intensity, as well as on spectral content. Consequently, if using Diff as features in classification, a small displacement can have a high influence on the identification performance. This effect does not exist in feature I, making it more robust to small changes in the position of the electrodes. On the other hand, the identification based on the combination of intensity and spatial features significantly outperforms both of them. This result was obtained both for identification of tasks and identification of tasks and effort levels. Furthermore, it has been shown that the classifier based on I + CG discriminates between types of tasks at low levels of effort (10 % MVC) significantly better than the classifiers based on the other feature sets (Fig. 5).

The impedance between electrodes and skin changes during time on account of several causes, e.g., drying of conductive gel and sweating. Consequently, the identification performance deteriorates as the time between the training of the classifier and the identification increases. When the identification is performed long after the training of classifier, the results show that the identification based on I + CG performs just slightly better than the identification based on I features, while the identification based on Diff features is much worse (S_I+CG_ = 94 %, P_I+CG_ = 95 %; S_I_ = 93 %, P_I_ = 94 %; S_Diff_ = 83 %, P_Diff_ = 83 %). Although it may seem that, in average, spatial features do not improve the classification with respect to using only the intensity of an HD-EMG map, it is important to outline that these results were obtained on contractions of high levels of effort (50 % MVC), where performances were similar even when contractions were recorded at the same time (see Fig. 5).

Muscle fatigue also affects the recorded EMG signal both in the time and spectral domains and therefore the identification performance deteriorates with fatigue. The results of this work show that the classifier based on intensity and spatial features is less sensitive to fatigue than classifiers based on the other feature sets. The proposed classifier shows a very good performance in task identification even at the final stage of fatigue (Acc = 91.3 %, S = 84.3 %, P = 87.0 %, SP = 93.5 %).

The proposed method could significantly improve the human-machine interface technology and can be used in numerous applications: computer games, exoskeletons, automatic wheelchairs, rehabilitation robots, prostheses, etc. As suggested by Müller-Putz et al. [44], non-invasive hybrid brain-computer interfaces (BCI) can be designed as EEG-based BCI supplemented with other biological and mechanical signals. For example, they reported significantly higher identification results for motion intention when using a hybrid BCI system composed of EEG and EMG sensory systems than when using only one of them. EMG usually has higher SNR ratio than EEG and it is widely used in the identification of the motion intention, however, it is prone to malfunction due to fatigue. When fatigue occurs, the supplemented EEG input keeps the identification stable, and increases the robustness of the system. Thus, advances in obtaining methods more robust to fatigue or time effect are very interesting.

Some patients with neuromuscular impairment can weakly activate their muscles, but insufficiently to generate a movement. In these patients, as well as in patients that can generate only weak movements, HD-EMG maps can be generated and used in identification of motion intention, as demonstrated in this study. This approach could supplement the existing BCI or inertial sensors based prostheses and result in a device with a better performance. For example, Rohm et al. [45] performed a very interesting study with a single SCI patient. Their neuroprosthesis consisted of a functional electrical stimulation of the forearm and upper arm muscles, and a semiactive elbow orthosis. Using BCI and a shoulder joystick, the patient was able to perform complex hand and elbow tasks from everyday life (e.g. eating an ice cream cone). The reported performance of that study was 70 %, which was remarkable considering the fact that the patient did not have any control over involved muscles. However, performance of similar patients could be increased using hybrid BCI if myoelectric activation exists.

Furthermore, compared to inertial signals, which are also used as input to control devices, EMG has a major advantage because myoelectric activation precedes the actual movement, which can save valuable response time.

However, it should be noted that although this study represents an improvement in the identification of motion intention, additional experiments should be considered in the future. Firstly, HD-EMG recordings were carried out during controlled isometric submaximal contractions, i.e. patient’s arm was fixed and supported by a mechanical brace. Since the methodology was capable to successfully and automatically differentiate between none, very low, low and medium effort levels, we might hypothesized that the method can be useful in prediction without the support of the brace. However, more experiments without the brace and the analysis of the recorded HD-EMG signals would be necessary to confirm and quantify this hypothesis.

## Conclusion

In this study, the spatial distribution of EMG intensity was evaluated for identification of tasks and different levels of effort in patients with iSCI. Results show that the spatial activation of motor units is dependent on the type of exercise and contraction intensity, and that related features can improve identification performance.

Although results show that spatial features also enhance the robustness of the identification to time effect and fatigue, additional experiments need to be performed to test robustness to temporal dependent changes more thoroughly and to determine when the classifier fails by further tests done on fatigue.

The center of gravity was used as a figure of merit to describe the spatial distribution. Although it shows a significant improvement in classification, by definition it is insensitive to fine changes in the distribution of muscle units. Therefore, in future works, more appropriate measures of spatial distribution should be analyzed in order to better describe the spatial distribution of muscle intensity. Also, additional features as those related to the frequency content could be considered to improve even more the classification performance.
